# Shifting paradigms in ICD tachytherapy deactivation in the final phase of life: eighteen years of perspective

**DOI:** 10.1093/ehjqcco/qcag038

**Published:** 2026-03-04

**Authors:** Ahmed A M al Jaff, Anastasia D Egorova, Dilek Yilmaz, Martin J Schalij, Lieselot van Erven

**Affiliations:** Department of Cardiology, Leiden Heart-Lung Center, Leiden University Medical Center, Albinusdreef 2, 2333 ZA, Postbus 9600, Leiden 2300 RC, The Netherlands; Department of Cardiology, Leiden Heart-Lung Center, Leiden University Medical Center, Albinusdreef 2, 2333 ZA, Postbus 9600, Leiden 2300 RC, The Netherlands; Department of Cardiology, Erasmus University Medical Center, Dr. Molewaterplein 40, 3015 GD, Rotterdam, The Netherlands; Department of Cardiology, Leiden Heart-Lung Center, Leiden University Medical Center, Albinusdreef 2, 2333 ZA, Postbus 9600, Leiden 2300 RC, The Netherlands; Department of Cardiology, Leiden Heart-Lung Center, Leiden University Medical Center, Albinusdreef 2, 2333 ZA, Postbus 9600, Leiden 2300 RC, The Netherlands

**Keywords:** Implantable cardioverter-defibrillator (ICD), Tachytherapy deactivation, Advance care planning, Palliative cardiology, Defibrillator shocks

## Abstract

**Aims:**

Implantable cardioverter-defibrillators (ICDs) may deliver painful shocks in the final phase of life. The European Heart Rhythm Association (EHRA) and Heart Rhythm Society (HRS) recommend advanced care planning and timely tachytherapy deactivation to prevent this. This study aims to evaluate anticipatory tachytherapy deactivation practices over an 18-year period and identify implementation gaps to explore strategies for improvement.

**Methods and results:**

Records of 1606 ICD or cardiac resynchronization therapy-defibrillator (CRT-D) patients (79% male; mean age 67 ± 10 years at implantation, 73 ± 10 at death) who died between 2006 and 2022 were reviewed. Heart failure (32%) and malignancy (15%) were the most common causes of death. Anticipatory tachytherapy deactivation occurred in 42% of patients. Deactivation rates increased 5.2% annually from 2006 to 2014 (*P* < 0.001), but plateaued from 2015 to 2022 (*P* = 0.403). Among deactivations, 33% occurred on the final day of life, mainly during hospitalization.

**Conclusion:**

Despite improvements in anticipatory tachytherapy deactivation practices, a plateau has been reached. Many deactivations still occur on the final day of life, underscoring the need for earlier and more proactive counselling. Integrating deactivation discussions into routine care, particularly for heart failure patients, will be essential to ensure patient-centered advanced care planning.

## Graphical Abstract

**Graphical Abstract:**
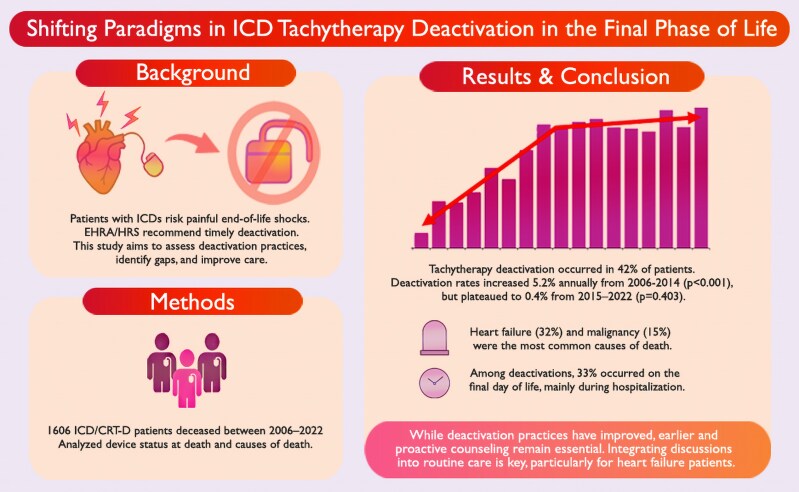

Key Learning Points What is already known

- ICDs may deliver painful shocks in the final phase of life, causing distress and prolonged suffering.
- EHRA/HRS expert consensus statements recommend timely tachytherapy deactivation.
- Although awareness has improved, there is limited data on how current real-world practice aligns with these recommendations.

What this study adds

- Tachytherapy deactivations increased significantly from 2006 to 2014, but plateaued afterwards.
- 33% of deactivations still occur on the final day of life, often in hospital settings, highlighting the need for earlier discussions.
- Integrating deactivation discussions into routine care, particularly for heart failure patients, will be essential to ensure patient-centred advance care planning.

## Introduction

Implantable cardioverter-defibrillators (ICDs) are a cornerstone therapy for primary prevention of sudden cardiac death (SCD) in high-risk patients and for secondary prevention in survivors of life-threatening ventricular arrhythmias.^1,2^ While ICDs significantly reduce arrhythmia-related mortality, most patients with ICDs eventually succumb to non-sudden death or even non-cardiac death.^3^ In the majority of these cases, the terminal phase may be anticipated, creating a window of opportunity for advance care planning and proactive tachytherapy management decisions. ICD shocks cause pain and anxiety in patients.^4,5^ Moreover, patients with active tachytherapy functions remain at risk of receiving shocks in the final hours of life, which can impose a high degree of discomfort and potentially prolong suffering for both patients and their families.^6,7^ When prolonging life and managing life-threatening arrhythmias is no longer the primary objective, tachytherapy deactivation should be considered and discussed with patients in a timely manner.^8,9^ In line with this, a European Heart Rhythm Association (EHRA) expert consensus statement advocating for the timely deactivation of tachytherapy prior to the last moments of life was published in 2010.^8^ This recommendation increased awareness and has contributed to a rise in timely tachytherapy deactivation across Europe.^1,3,10^

However, there is a paucity of data on how contemporary real-world practice aligns with these recommendations. This study aims to evaluate the temporal changes in tachytherapy deactivation practice over the past 18 years, identify scenarios and patient characteristics associated with suboptimal implementation of the 2010 EHRA consensus statement recommendations, in order to define strategies to enhance anticipatory ICD tachytherapy management prior to the last moments of life.

## Methods

### Study design

**Figure 1:**
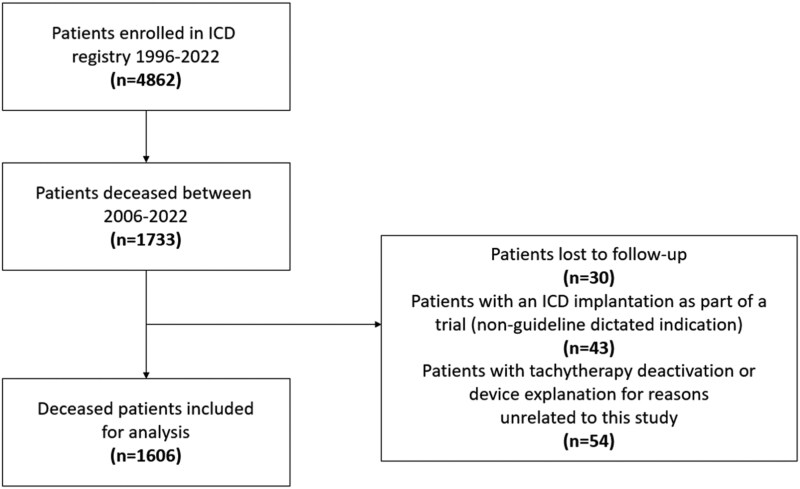
Diagram of patient selection. A total of 4862 patients from the ICD registry (1996–2022) were screened. Of these, 1733 died between 2006 and 2022, and data of 1606 patients were included for analysis. Exclusion reasons were: lost to follow-up (*n* = 30), initial ICD implantation as part of a clinical trial (non-guideline dictated indications (*n* = 43) and tachytherapy deactivation or device explantation due to expiration of ICD-indication (*n* = 54).

This retrospective cohort study included all patients who underwent an ICD or Cardiac Resynchronization Therapy-Defibrillator (CRT-D) implantation at the Leiden University Medical Center (LUMC) between January 1996 and December 2022 as part of an ongoing prospective registry. Patients who died between 1 January 2006, and 31 December 2022 were screened for inclusion, given the availability of comprehensive digital follow-up data in that period. Patients were excluded if they were followed up at another centre, were lost to follow-up for 12 months or longer, or if tachytherapy was deactivated due to expiration of ICD-indication. A study flowchart is shown in *Figure 1*.

### Data collection

Demographic and clinical data were collected from the hospital’s electronic patient records—EPD Vision (Leiden, the Netherlands) and HiX (Chipsoft, Amsterdam, the Netherlands). Following device implantation, patients underwent scheduled device interrogation every six months (or more frequently if clinically indicated) and had at least one annual follow-up consultation with a cardiologist. Any modifications to ICD/CRT-D settings were documented in a dedicated section of the electronic records, in accordance with hospital protocol.

Survival status and date of death were obtained from municipal civil records. Patient records were systematically reviewed to determine the circumstances and causes of death, as well as the ICD tachytherapy settings at the time of death. In cases where tachytherapy was deactivated prior to death, both the initiator of deactivation and the device settings were recorded. For hospitalized patients, the cause of death was adjudicated based on hospital records. In case of missing documentation, the cause of death was retrieved from the general practitioner’s records.

#### Outcome definitions

Causes of death were classified into three groups according to the Hinkle–Thaler Classification: cardiac death, non-cardiac death, and sudden death.^12^ For the purposes of this study, cardiac and non-cardiac deaths were further subdivided to capture more detailed outcomes, particularly regarding tachytherapy deactivation and care for patients in the palliative phase. In cases involving palliative sedation and euthanasia, mode of death was categorized according to the underlying condition to ensure consistency.

Cardiac death was subdivided in heart failure death, defined as death due to terminal heart failure or progressive failure of cardiac pump function and other cardiac causes, such as myocardial infarction, and valvular disease complications.^13^ Non-cardiac death was subdivided into the malignancy, infection/sepsis, stroke/neurodegenerative disease and other non-cardiac causes such as respiratory disease or renal failure. Sudden death was defined as unexpected death without any prior documented clinical deterioration. Deaths due to documented tachyarrhythmia were also included in this category. If a patient had died suddenly but an identifiable cause was present, the death was classified as a non-sudden case and adjudicated to that specific category for analysis purposes.

### Ethics statement

All tests and procedures involving human participants were in accordance with the ethical standards of the institutional and/or national research committee and with the 2013 Helsinki Declaration. Appropriate local scientific board approval was obtained for the protocol (2023-020) and the need for written informed consent was waived by the Medical Research Ethics Committee Leiden Den Haag Delft due to retrospective study design and data anonymity.

### Statistical analysis

All statistical analyses were performed in SPSS version 25 (IBM Corp, Armonk, NY, USA). Based on their distributions, normally distributed data are presented as mean ± standard deviation and not normally distributed data as median with interquartile ranges [IQR1-IQR3], unless stated otherwise. Assessment of normal distribution was done visually and tested using the Kolmogorov–Smirnov and Shapiro–Wilk tests. Comparison of normally distributed data was done using a Student’s *t*-test, while non-normally distributed data was compared using the Mann–Whitney *U* test. Proportional differences were compared using Chi-square (*χ*^2^) analysis or Fisher’s exact test, as appropriate. To evaluate trends in deactivation rates over time, a spline regression analysis was performed. Predicted values from the regression model were used to illustrate trends. A two-sided *P*-value ≤0.05 was considered statistically significant.

## Results

### Patient characteristics at time of ICD implantation

A total of 4862 consecutive patients were enrolled in the Leiden ICD implantation registry in the period between 1996 and 2022. Of these, 1733 patients died in the time between 2006 and 2022. After excluding 127 patients for prespecified reasons, 1606 patients were included for analysis, *Figure 1*.

Patient characteristics at the time of device implantation are summarized in *Table 1*. The majority of patients were male (*n* = 1267, 79%), and the mean age at implantation was 67 ± 10 years. Primary prevention was the indication in 1083 patients (67%), with 393 (24%) having received an ICD and 690 (43%) a cardiac resynchronization therapy defibrillator (CRT-D). Ischaemic cardiomyopathy was the most prevalent underlying aetiology (*n* = 1094, 68%). The median left ventricular ejection fraction (LVEF) was 30% [22–37]. Most patients were in the New York Heart Association (NYHA) Class II (*n* = 586, 37%) or III (*n* = 567, 36%).

**Table 1:** Patient characteristics at time of ICD implantation

Baseline characteristic	ICD cohort (*n* = 1606)	Active tachytherapy (*n* = 935)	Deactivated tachytherapy (*n* = 671)	*P* value
Male; sex–no. (%)	1267 (79%)	745 (80%)	522 (78%)	0.361
Age at implantation–years (+/− SD)	67 ± 10	67 ± 10	67 ± 10	0.866
Body-mass index kg/m^2^ (+/− SD)	26.5 ± 13.8	27.01 ± 15.2	25.8 ± 11.7	0.083
Indication for ICD–no. (%)				
Primary prevention	393 (24%)	221 (24%)	172 (26%)	0.379
Secondary prevention	420 (26%)	249 (27%)	171 (25%)	
Indication for CRTD — no. (%)				
Primary prevention	690 (43%)	398 (42%)	292 (44%)	0.157
Secondary prevention	103 (7%)	67 (7%)	36 (5%)	
Underlying cardiac condition				
Ischemic cardiomyopathy — no. (%)	1094 (68%)	663 (70%)	431 (64%)	0.005
Non-ischemic cardiomyopathy no (%)	449 (28%)	240 (26%)	209 (31%)	0.332
Congenital Heart Disease — no. (%)	26 (2%)	15 (2%)	11 (2%)	0.958
Electrical heart disease — no. (%)	37 (2%)	17 (2%)	20 (3%)	0.770
Left ventricular ejection fraction—% [Q1-Q3]	30 [22–37]	30 [21–38]	30 [22–37]	0.351
NYHA–no. (%)				
Class I	362 (23%)	197 (22%)	165 (25%)	0.250
Class II	586 (37%)	337 (37%)	249 (38%)	
Class III	567 (36%)	343 (37%)	224 (34%)	
Class IV	60 (4%)	38 (4%)	22 (3%)	
Creatinine in mmol/L [Q1-Q3]	101 [83–126]	102 [84–128]	100 [82–124]	0.042
Comorbidities/history				
Hypertension–no. (%)	691 (43%)	387 (41%)	304 (45%)	0.147
Diabetes–no. (%)	421 (26%)	279 (30%)	142 (21%)	<0.001
COPD–no. (%)	191 (12%)	122 (13%)	69 (10%)	0.089
CVA–no. (%)	178 (11%)	107 (11%)	71 (11%)	0.567

### Patient characteristics at time of death

**Figure 2:**
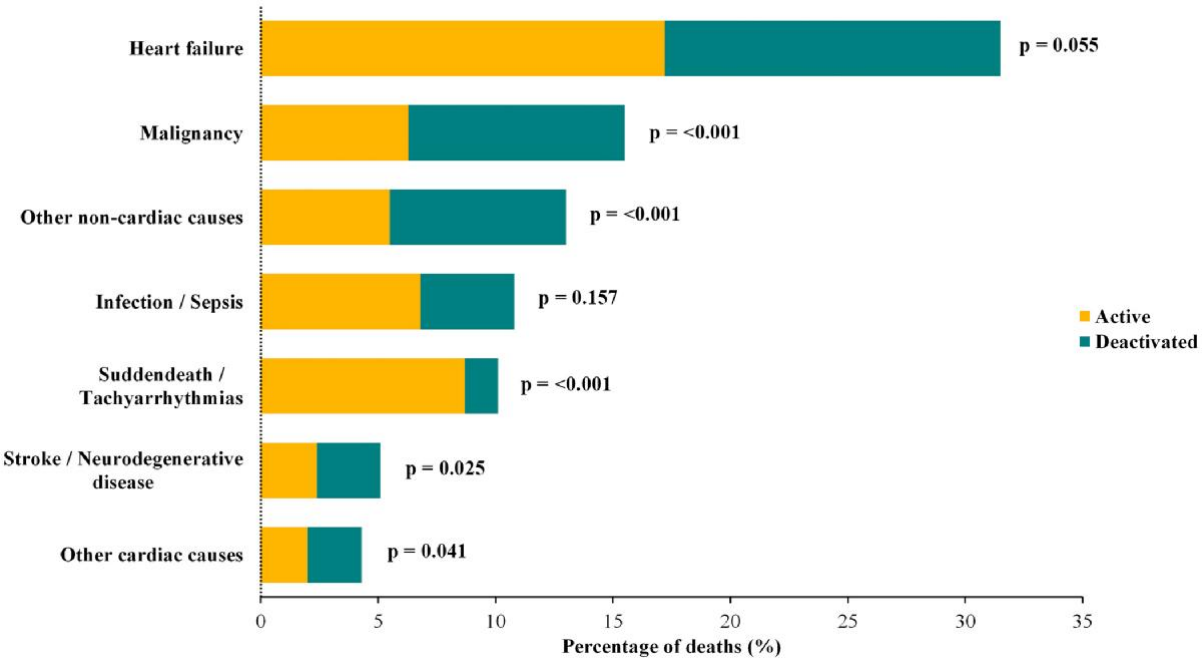
Tachytherapy deactivation status at time of death stratified per mode of death. The percentage of deaths in the total cohort categorized by cause of death and tachytherapy deactivation status. The patients with an unknown/undetermined (*n* = 157, 10% of the total cohort) cause of death were excluded from this analysis.

At the time of death, the mean age was 73 ± 10 years. The median years of active tachytherapy were 5.7 years [2.8–9.6]. Pulse generator replacements occurred in 746 patients (47%), with 477 (30%) having undergone 1 replacement procedure, 192 (12%) having undergone 2, and 77 (5%) having undergone ≥ 3 replacement procedures during their lifetime, *Table 2*. Of interest, 173 patients (11%) had undergone a device replacement procedure in their last year of life. Overall, therapy for ventricular arrhythmias had been delivered in 700 patients (44%), of which 127 (8%) received only shocks, 241 (15%) received only anti-tachycardia pacing (ATP), and 332 (21%) received both at some point in their lives. The most common cause of death was heart failure (*n* = 506, 32%), followed by malignancies (*n* = 248, 15%) and infection/sepsis (*n* = 174, 11%), *Figure 2*.

**Table 2:** Patient characteristics at time of death

Patient characteristic	Total cohort (*n* = 1606)	Active (935)	Deactivated (671)	*P* value
Age at death (years) (+/− SD)	73 ± 10	72 ± 10	74 ± 10	<0.001
Years of active tachytherapy **—** median years [Q1-Q3]	5.7 [2.8–9.6]	5.2 [2.3–8.4]	6,5 [3.5–10.8]	<0.001
Device replacement–no. (%)	746 (47%)	415 (44%)	331 (49%)	0.050
Cumulative number of pulse generator replacements–no. (%)				
1	477 (30%)	282 (30%)	195 (29%)	0.634
2	192 (12%)	95 (10%)	97 (14%)	0.009
≥3	77 (5%)	38 (4%)	39 (6%)	0.106
No appropriate therapy during lifetime–no. (%)	906 (56%)	512 (55%)	394 (59%)	0.126
Appropriate therapy during lifetime–no. (%)				
Shocks	127 (8%)	85 (9%)	42 (6%)	0.039
ATP	241 (15%)	142 (15%)	99 (15%)	0.832
ATP and shocks	332 (21%)	196 (21%)	136 (20%)	0.755
Cause of death–no. (%)				
Heart failure	506 (32%)	277 (30%)	229 (34%)	0.055
Malignancy	248 (15%)	101 (11%)	147 (22%)	<0.001
Infection/sepsis	174 (11%)	110 (12%)	64 (9%)	0.157
Sudden death/tachyarrhythmias	162 (10%)	139 (15%)	23 (3%)	<0.001
Stroke/neurodegenerative disease	82 (5%)	38 (4%)	44 (7%)	0.025
Other cardiac causes	69 (4%)	32 (3%)	37 (6%)	0.041
Other non-cardiac causes	208 (13%)	88 (9%)	120 (18%)	<0.001
Unknown	157 (10%)	150 (16%)	7 (1%)	<0.001

Years of active tachytherapy were measured from the time of ICD implantation to death for patients with active tachytherapy. For patients with deactivated tachytherapy, duration was measured from implantation to the time of deactivation.

### Temporal changes in ICD therapy deactivated prior to the time of death (2006–2022)

**Figure 3:**
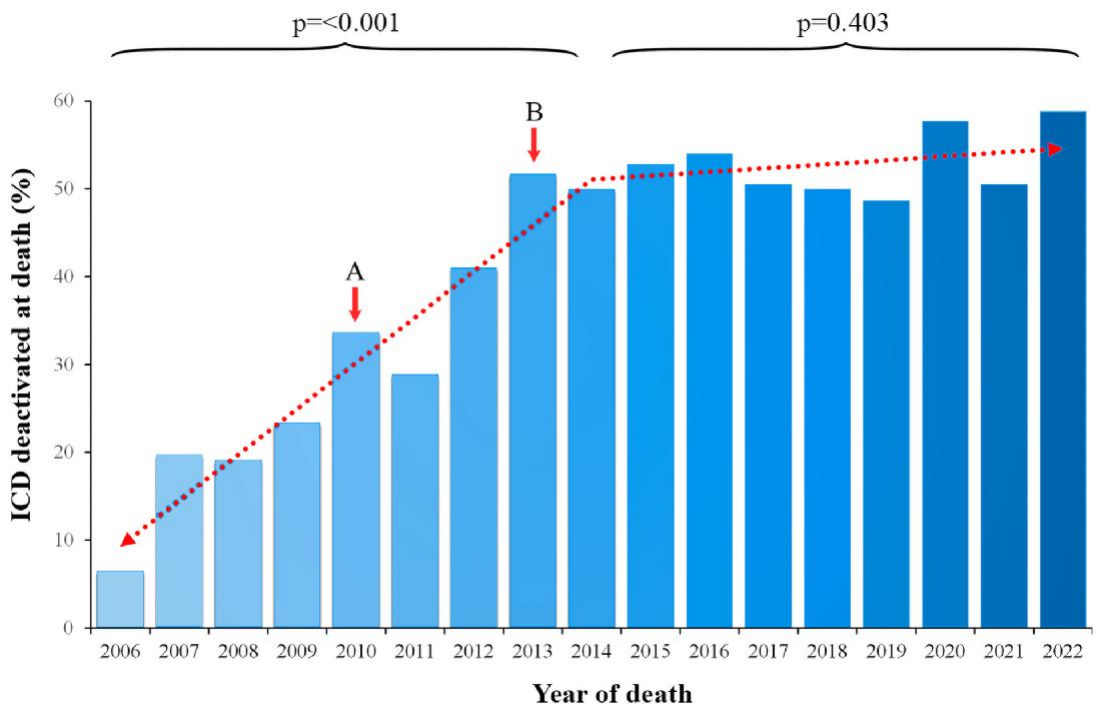
Temporal changes in the proportion of patients with ICD therapy deactivated prior to the time of death (2006–2022). (*A*): The publication of the European Heart Rhythm Association^8^ and American Heart Rhythm Society^9^ consensus statements (2010). (*B*): The publication of the Dutch guidelines (2013).^27^ Spline regression analysis was performed with a knot in the year 2014. Two distinct slopes were calculated. The first spline (2006–2014) had a slope of 5.2% per year, which was statistically significant (*P*=<0.001). The second spline (2015–2022); had a slope of 0.4% per year, which was not statistically significant (*P* = 0.403). The red dotted trendline shows the rate of the predicted value based on the spline regression model.

A gradual increase in the anticipatory ICD deactivation was observed until 2014 and levelled off afterwards. In the year 2006, the deactivation rates were 7%, while in 2022, this was 59%. Based on predicted values from a spline regression, the slope of increase from 2006 to 2014 was 5.2% per year (*P* < 0.001), while in the years from 2015 to 2022 the slope did not have a significant increase (0.4%, *P* = 0.403). Further information regarding the changes in deactivation is depicted in *Figure 3*.

### Timing and location of tachytherapy deactivation

**Figure 4:**
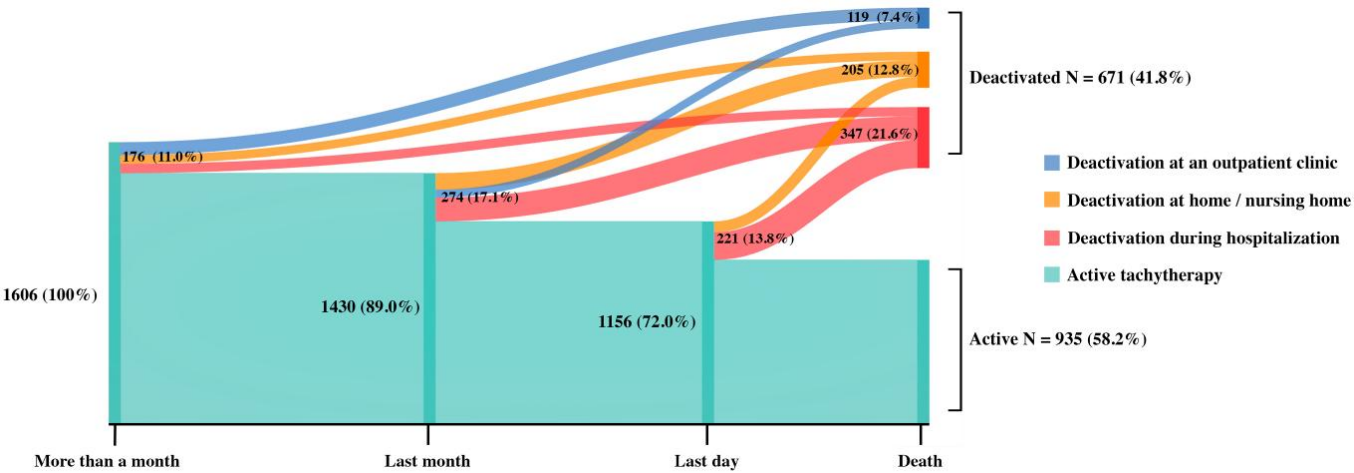
Timing and location of ICD tachytherapy deactivation in relation to death. This Sankey diagram illustrates the timing of anticipatory tachytherapy deactivation, categorized as occurring more than a month prior to death, during the last month, or on the last day of life. The deactivations are further stratified by location: during hospitalization, outpatient clinic, or at home/nursing home.

Of the 671 patients with anticipatory tachytherapy deactivation, 176 (26%) were deactivated more than one month before death, primarily at an outpatient clinic (*n* = 74, 11%). In 274 patients (41%), deactivation occurred within the last month of life, most commonly during hospitalization, (*n* = 135, 20%) or at home/nursing homes (*n* = 94, 14%). Deactivations on the last day of life were reported in 221 patients (33%), occurring either during hospitalization (*n* = 159, 24%) or at home/nursing homes (*n* = 62, 9%), *Figure 4*.

### Demographic and characteristic differences regarding tachytherapy deactivation

Patients who underwent tachytherapy deactivation prior to death were significantly older than those with active tachytherapy (74 ± 10 years vs. 72 ± 10 years, *P* < 0.001). The median duration for which patients had active therapy was also significantly longer in the deactivated group (6.5 years [3.5–10.8] vs. 5.2 years [2.3–8.4], *P* < 0.001). Patients with ischaemic cardiomyopathy were more likely to die with active tachytherapy than with deactivated tachytherapy functions (70% [*n* = 663] vs. 64% [*n* = 431]; *P* = 0.005). Anticipatory deactivation was significantly more common among patients who died of malignancy (*n* = 147, 22% vs. *n* = 101, 11%, *P* < 0.001). In contrast, among patients who died from sudden death/tachyarrhythmia, significantly more had active tachytherapy at the time of death (*n* = 139, 15% vs. *n* = 23, 3%, *P* < 0.001). Although heart failure was the most prevalent cause of death in the overall cohort, there was no correlation with anticipatory tachytherapy deactivation, *Figure 2*.

## Discussion

This study provides a comprehensive evaluation of tachytherapy deactivation practices over 18 years, highlighting temporal trends, evolving clinical practices as well as persisting implementation gaps. The key findings are: (i) anticipatory tachytherapy deactivation occurred in 42% of patients. The tachytherapy deactivation rates increased significantly over time, showing an annual rise of 5.2% between 2006 and 2014; however, from 2015 to 2022, this trend plateaued. (ii) Among patients who underwent tachytherapy deactivation, 41% had it deactivated within the last month of life, and 33% on the final day, both predominantly during hospitalization. This suggests that the decision for deactivation is often made under time-constrained conditions. (iii) Although heart failure was the most prevalent cause of death, it was not significantly associated with higher rates of tachytherapy deactivation.

### Shocks at the end of life

There is limited data on the burden of ventricular arrhythmias in ICD patients during the final phases of life. Post-mortem pre-explant read-outs are not standard practice and data are difficult to obtain, particularly when patients die outside the hospital. The only prospective study on this topic, for which devices of deceased patients in a Swedish centre were explanted and systematically analysed, revealed that 35% of the patients experienced a ventricular tachycardia episode in the last hour of their lives.^6^ However, since 82% of the patients in this study had ICDs for secondary prevention, these results may not be directly applicable to our cohort, where primary prevention indication was more prevalent. Shocks in the last phase of life are often perceived as distressing, affecting not only the patients but also their families and healthcare providers.^14^ This underscores the importance of anticipatory tachytherapy deactivations as part of advance care planning.

### Tachytherapy deactivation in heart failure patients

Choosing the appropriate moment for tachytherapy deactivation in patients with heart failure can be particularly challenging.^14^ Unlike malignancies, which often follow a more predictable terminal trajectory, heart failure is frequently marked by recurring exacerbations and hospitalizations.^15^ Given the complexity of managing heart failure patients, the cardiologist plays a central role in determining continuation of intensive treatment. Still, from our data, it appears that identifying the optimal timing for tachytherapy deactivation in heart failure is difficult, since only in about half of the heart failure patients, tachytherapy was deactivated. Notably, one in six patients hospitalized for heart failure die within 30 days after the index hospitalization, indicating that hospitalization might represent a valuable opportunity to initiate advanced care planning.^16^ Early discussions about tachytherapy deactivation, particularly following hospital admissions, can help in ensuring that patients have adequate time to make well informed and contemplated decisions.

### Barriers to tachytherapy deactivation

Previous studies have shown that various factors influence a patient’s decision regarding tachytherapy deactivation. Avoidance of painful shocks, and experiencing a higher frequency of shocks are among a key factors for patients with a favourable attitude towards tachytherapy deactivation.^17,18^ Religious and cultural beliefs may also play a significant role in decision-making. According to Raphael *et al.*, approximately 40% of patients felt that their religious beliefs were important when contemplating tachytherapy deactivation.^17^ Furthermore, understanding the patients’ perceptions about the function and benefits of tachytherapy is crucial, as heart failure patients often overestimate its life-prolonging benefit.^19^

Barriers to tachytherapy deactivation discussions and implementations apply not only to patients, but also to healthcare professionals. Clinicians may face challenges such as limited experience or knowledge, difficulty predicting disease trajectory, and time constraints.^20^ These barriers, particularly time constraints, may be more pronounced when caring for patients with multiple comorbidities, where limited consultation time can reduce opportunities to address anticipatory management topics like tachytherapy deactivation.^21^ This may partly explain why, in our study, patients with diabetes mellitus were significantly less likely to undergo tachytherapy deactivation.

### Tachytherapy deactivation throughout Europe

Since the publication of the EHRA consensus statement in 2010, awareness of tachytherapy deactivation has increased throughout Europe.^3,10,11,22^ In Sweden, an increase in tachytherapy deactivations was observed, particularly among patients dying at the cardiology ward, though this trend was not observed for other specialties.^10^ Similarly, in Northern Ireland, discussions about deactivations were documented in up to 52% of patients, corresponding to a deactivation rate of 36%.^11^ Throughout different centres in the Netherlands, a notable rise was observed.^3,22^ In our centre, the publication of the European guidelines published in 2010 was associated with a 10% increase in deactivation rates in the following year, while in 2013, the publication of the Dutch guidelines was associated with a further 11% increase. One should be cautious regarding data extrapolation to other countries. The Netherlands is a relatively small and densely populated country in which deactivation at patients’ homes or nursing homes can be facilitated on relatively short notice. In countries with different geographical variations and healthcare systems, this might be more challenging.

### Future perspectives

Moving forward, greater emphasis should be placed on identifying patients who are less likely to undergo tachytherapy deactivation and initiating timely discussions. In a study by Januszkiewicz *et al.*, only 25% of patients were aware at the time of implantation that tachytherapy deactivation could be considered in the final phase of life. This underscores a major gap in the information provided to patients, especially since the period prior to implantation is regarded as the second-best moment by ICD patients to initiate discussions about tachytherapy deactivation. The optimal time remains when a patient’s health significantly deteriorates.^23^ Recognizing this gap, healthcare professionals can seize timely opportunities to initiate these discussions, which is particularly relevant for heart failure patients. Initiating informative deactivation conversations, particularly during the first heart failure-related hospitalization, can be beneficial, especially when considering that 1 in 6 patients reportedly dies in 30 days after the first hospitalization.^16^ Integrating discussions on tachytherapy deactivation into routine clinical pathways will be essential to ensure timely and effective advance care planning. Many ICD centres employ specialized ICD nurses who are actively involved in all aspects of ICD care; incorporating these conversations into their routine may facilitate a more structured and consistent approach for future care.^24^ Alongside increased attention to anticipatory tachytherapy deactivation, more focus should also be given to refraining from unnecessary device replacements in patients in the nadir of death. In our study, 173 patients (10.8%) underwent a device replacement procedure in their last year of life. Identifying patients at high risk of mortality within 1 year of device replacement has become increasingly important, and efforts to improve individual risk stratification are ongoing.^25,26^ Moreover, initiating discussions with patients approximately one year prior to replacement may provide an appropriate opportunity to address the option of tachytherapy deactivation, particularly in patients with characteristics associated with a high one-year mortality risk.^26^

### Limitations

Consecutive patients were enrolled in the ICD registry during several decades; evolving guidelines and best practices may have contributed to a heterogeneous population of ICD and CRT-D carriers, potentially influencing the trends observed over time. Furthermore, some patients may have died under the care of different healthcare providers, potentially resulting in an underestimation of the deactivation rates. The inclusion of data regarding tachytherapy during the last days of life would provide valuable insights; however, post-mortem device readouts are not standard practice at our centre, limiting the availability of such data. This was further limited by the high proportion of patients who died in nursing homes or other non-hospital settings, where device readout is logistically challenging. Similarly, information regarding the location of death in patients with active tachytherapy was limited and, therefore, excluded. Finally, the retrospective and non-randomized nature of this study prevents us from drawing causal inferences. Nevertheless, the large patient numbers and the granularity of the data provide us with important insights into associations and temporal changes.

## Conclusions

Despite improvements in anticipatory tachytherapy deactivation practices, a plateau has been reached. Many deactivations still occur on the final day of life, underscoring the need for earlier and more proactive counselling. Integrating deactivation discussions into routine care, particularly for heart failure patients, will be essential to ensure patient-centred advanced care planning.

## Data Availability

The data presented in this study are available upon reasonable request from the corresponding author. The data are not publicly available due to privacy restrictions.
